# The role of lipoprotein (a) in chronic kidney disease

**DOI:** 10.1194/jlr.R083626

**Published:** 2018-01-29

**Authors:** Jemma C. Hopewell, Richard Haynes, Colin Baigent

**Affiliations:** Clinical Trial Service Unit & Epidemiological Studies Unit,* Nuffield Department of Population Health, University of Oxford, Oxford, United Kingdom; Medical Research Council Population Health Research Unit,† Oxford, United Kingdom

**Keywords:** atherosclerosis, epidemiology, renal disease

## Abstract

Lipoprotein (a) [Lp(a)] and its measurement, structure and function, the impact of ethnicity and environmental factors, epidemiological and genetic associations with vascular disease, and new prospects in drug development have been extensively examined throughout this Thematic Review Series on Lp(a). Studies suggest that the kidney has a role in Lp(a) catabolism, and that Lp(a) levels are increased in association with kidney disease only for people with large apo(a) isoforms. By contrast, in those patients with large protein losses, as in the nephrotic syndrome and continuous ambulatory peritoneal dialysis, Lp(a) is increased irrespective of apo(a) isoform size. Such acquired abnormalities can be reversed by kidney transplantation or remission of nephrosis. In this Thematic Review, we focus on the relationship between Lp(a), chronic kidney disease, and risk of cardiovascular events.

Lipoprotein (a) [Lp(a)] is synthesized in the liver and is comprised of a single LDL particle linked to a highly polymorphic apo(a) protein (1). Plasma Lp(a) levels vary widely between individuals, from 0 to >200 mg/dl, and have a highly skewed distribution resulting in most Europeans having levels <10 mg/dl (2). Blood levels of Lp(a) are highly heritable and are chiefly determined by copy number variation at the *LPA* locus on chromosome 6, as well as by ethnically determined differences (3). Copy number variation in the kringle IV type 2 protein domain, encoding apo(a) isoforms of differing size, results in a varying number of copies of identical kringle IV repeats. Lp(a) levels are inversely correlated with the genetically determined apo(a) isoform size, with higher levels of plasma Lp(a) being associated with smaller apo(a) isoform sizes as represented by fewer kringle IV repeats. Lp(a) levels can also vary considerably among individuals with a given apo(a) isoform size (4).

Epidemiological and genetic studies have suggested that raised Lp(a) levels are associated with, and have a causal role in, coronary heart disease and calcific aortic valve disease such that those with higher plasma levels of Lp(a) have a higher risk of disease (5–7). Consequently, Lp(a) is a potential target for which treatments are currently under development.

Kidney disease is associated with both an increased risk of vascular disease and an acquired elevation in Lp(a) levels. Furthermore, increases in Lp(a) levels in patients with kidney disease appear to be dependent on apo(a) isoform size. In this review, we will summarize the complex inter-relationships between Lp(a) levels and kidney function, and we will consider the insight this can offer into Lp(a) metabolism, the potential importance of Lp(a) for risk of vascular disease in patients with chronic kidney disease (CKD), and whether novel Lp(a)-lowering therapies might have a role in preventing cardiovascular disease in such patients.

## CHRONIC KIDNEY DISEASE

CKD is defined as the presence of kidney damage (manifesting as albuminuria, or determined by radiological or histological evidence) or decreased kidney function [glomerular filtration rate (GFR) <60 ml/min/1.73 m^2^] for at least 3 months. CKD can be characterized by its cause, stage (defined by GFR), and the level of albuminuria, with worse GFR and albuminuria each independently predicting a worse renal prognosis (8). CKD is common and affects 10–15% of most populations, but only about 0.1% have the most severe form, i.e., end-stage renal disease (ESRD), defined as the need for dialysis or kidney transplantation (9).

Some individuals with high levels of albuminuria have “nephrotic range” albuminuria (urine albumin:creatinine ratio >220 mg/mmol). If this is accompanied by low serum albumin and edema, it is termed the “nephrotic syndrome” and is always caused by damage to the glomerulus. The nephrotic syndrome is also associated with a high risk of venous and arterial thrombosis (in part due to loss of anticoagulant factors in the urine), infection (due to loss of immunoglobulins in the urine), and a marked hypercholesterolemia (with plasma total cholesterol concentrations typically over 10 mmol/l).

Diabetes is the leading cause of CKD in many countries. Other causes include chronic glomerular or tubulointerstitial diseases, structural abnormalities, and inherited conditions like polycystic kidney disease. CKD is also associated with older age, hypertension, and obesity (10). Furthermore, low estimated GFR (eGFR) and high albuminuria are both associated with increased risk of cardiovascular events (11). For example, a meta-analysis of observational studies has suggested that each 30% reduction in eGFR is associated with a 30% increase in risk of major vascular events (12).

## Lp(a) LEVELS IN CKD

Plasma Lp(a) levels reflect a balance of Lp(a) synthesis, which occurs in the liver, and catabolism, which is thought to involve the kidney but is less clearly understood (13–21). Higher Lp(a) levels have been observed with reduced eGFR, even at the earliest stages of renal impairment (22–27). In the Penn Diabetes Heart Study based on 1,852 patients with type 2 diabetes [but no evidence of clinical cardiovascular disease or poor kidney function (eGFR <60 ml/min/1.73 m^2^)], higher Lp(a) levels were associated with mild kidney impairment (eGFR 60–90 ml/min/1.73 m^2^) even after adjustment for albuminuria (25). A population study involving 7,675 individuals from different ethnic backgrounds also reported that lower eGFR (based on eGFR categories of 15–59, 60–89, 90–149, and >150 ml/min/1.73 m^2^) was weakly positively associated with serum Lp(a) levels, particularly in non-Hispanic blacks, suggesting potential ethnic differences (26). However, given that Lp(a) levels for a fixed apo(a) isoform size may vary between different ethnic groups, unmeasured apo(a) isoform size differences in this report may explain some of the observed ethnic heterogeneity. In one of the most detailed studies to date, Kronenberg et al. (27) examined the relationship between kidney function and apo(a) isoform size as well as Lp(a) levels in 227 non-nephrotic Caucasian patients with differing degrees of renal impairment. Lp(a) concentrations were significantly higher in those with renal disease when compared with 227 age-, gender-, and apo(a) phenotype-matched Caucasian controls [creatinine of 2.02 mg/dl (SD 1.16) in patients with CKD versus 0.99 mg/dl (SD 0.18) in controls] even when the GFR was not yet abnormal (i.e., GFR >90 ml/min/1.73 m^2^). Furthermore, kidney function was inversely correlated with Lp(a) levels, with those with the worst kidney function having highest Lp(a) levels, independent of the type of primary renal disease. However, the relationship between Lp(a) levels and kidney function was only seen in the subgroup of those non-nephrotic patients with large apo(a) isoforms, among whom median Lp(a) levels varied from 6.2 mg/dl in individuals with GFR >90 ml/min/1.73 m^2^ to 18 mg/dl in those with GFR <45 ml/min/1.73 m^2^ (27). Despite the mounting evidence of Lp(a) abnormalities in CKD, not all studies have replicated the association between Lp(a) and reduced GFR. For example, there was no significant association between Lp(a) and GFR in 804 individuals with stage 3–4 CKD (with GFR ranging from 13 to 55 ml/min/1.73 m^2^) and no suggestion of an interaction with apo(a) isoform size (28). In addition, a study of 87 kidney donors whose average kidney function was reduced from an eGFR of 112 before donation to 72 ml/min/1.73 m^2^ 1 year later, showed no significant difference in Lp(a) as a result of donation (18 mg/dl before donation versus 19 mg/dl 1 year after donation, *P* = 0.07), albeit apo(a) isoform size was not available to examine associations specifically in those with large apo(a) isoforms (29).

Numerous studies have also examined Lp(a) levels in patients with more severe CKD (i.e., ESRD), as well as the influence of dialysis modality. ESRD patients undergoing hemodialysis have been shown to have increased Lp(a) levels compared with healthy controls (30–41), and patients on regular hemodialysis to have five to ten times higher levels of Lp(a) compared with patients with early stage CKD (36). Similarly to the non-nephrotic CKD patients discussed above, Lp(a) elevations in ESRD patients treated with hemodialysis are apo(a) isoform size specific, with only those with large apo(a) isoforms showing higher Lp(a) levels than healthy controls (22, 30, 39–41). This association was illustrated in a study comparing 138 patients treated with hemodialysis and 236 controls in which, despite no difference in apo(a) isoform size frequency, only patients with large apo(a) isoforms showed 2- to 4-fold higher Lp(a) levels (39). By contrast, studies have shown that ESRD patients undergoing continuous ambulatory peritoneal dialysis (peritoneal dialysis) have higher Lp(a) levels independent of apo(a) isoform size (22, 30, 42). Milionis et al. (22) compared 47 patients undergoing peritoneal dialysis and 79 patients undergoing hemodialysis with healthy controls and found higher Lp(a) levels only in those treated with hemodialysis who had large apo(a) isoforms, but increased Lp(a) in peritoneal dialysis patients irrespective of apo(a) isoform size. **Table 1** summarizes the associations of CKD and dialysis modalities on Lp(a) levels.

**TABLE 1.**
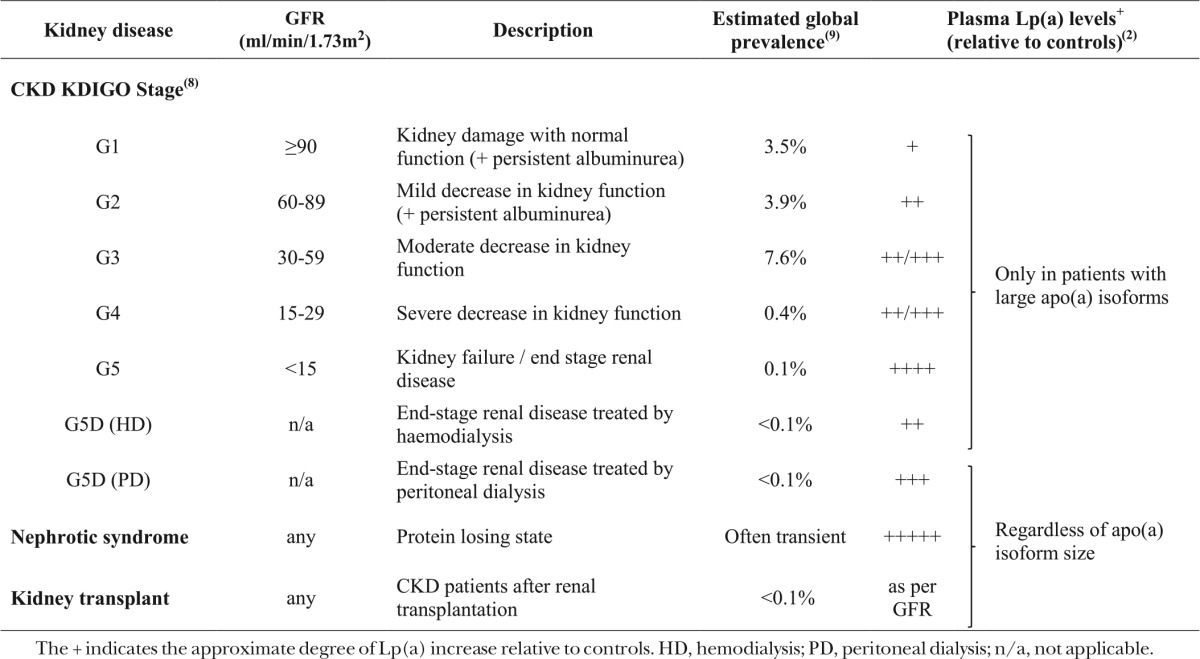
Summary of the effects of CKD and its management on Lp(a) levels

The acquired Lp(a) abnormality in patients with CKD appears to be the result of decreased Lp(a) clearance. An in vivo turnover study of stable isotopes in hemodialysis patients has suggested that the fractional catabolic rates of the Lp(a) protein components, Lp(a)-apo(a) and Lp(a)-apoB, are significantly lower in these patients than in healthy controls, thereby resulting in prolonged residence times; while by contrast, the production rates do not differ (15). This suggests that the higher Lp(a) levels observed in hemodialysis patients are the result of a decrease in Lp(a) clearance rather than an increase in production. The role of the kidney in clearance is further supported by several studies (15–17, 21, 43). Kronenberg et al. (21) have demonstrated lower Lp(a) levels in the renal vein than in the ascending aorta in patients undergoing coronary angioplasty, suggesting that Lp(a) is removed by the renal circulation. A study comparing 55 patients with CKD (creatinine clearance <70 ml/min) and matched controls found that urinary apo(a) was lower among patients with CKD (44), suggesting that the kidney may be involved in clearing intact apo(a) from the circulation, in addition to clearing apo(a) fragments produced by proteases in various tissues and pre-renal degradation (45). Small apo(a) particles [by contrast with larger apo(a) particles] may be directly secreted intact into the urine (45), so as kidney function declines, this secretion falls and plasma concentration of small apo(a) particles increases. However, while urinary apo(a) secretion positively correlates with plasma Lp(a) levels, the overall fraction of Lp(a) cleared by the kidney is likely to be only a small proportion of total apo(a) catabolism (43).

While it is clear that reduced kidney function influences Lp(a) levels and its catabolism, it is also possible that Lp(a) has a causal role in renal disease progression. A prospective study of 862 diabetic patients suggested that Lp(a) was an independent prognostic factor for the development of CKD in type 2 diabetes (46). In the CRIC study of 3,939 adults with CKD, however, about half of whom had diabetes, Lp(a) at baseline was not significantly associated with subsequent progression of kidney disease (defined as a 50% decline in eGFR or progression to ESRD) (47). In support of a role of Lp(a) in the development of CKD, Emdin et al. (48) reported that an *LPA* genotype score, associated with lower Lp(a) levels, was nominally associated with a lower risk of CKD [odds ratio, 0.91; 95% CI, 0.83–1.00; *P* = 0.04 per SD (∼28 mg/dl) lower genetically determined Lp(a)]. Furthermore, the *LPA* genotypes were also associated with ∼2 ml/min improvement in eGFR (*P* = 1.4 × 10^−5^), consistent with a previously reported effect of *LPA* on creatinine levels (49). This potentially implicates Lp(a) metabolism in CKD development, but further work is needed to better understand this relationship and its potential impact given the wider complex interrelationship between Lp(a) and kidney disease, and more data are needed to clarify the role of Lp(a) in kidney disease progression. Furthermore, the impact of any confounding with concomitant vascular disease remains unclear.

## Lp(a) LEVELS IN PROTEIN-LOSING STATES

Lp(a) levels are raised in renal disorders associated with protein loss, and patients with the nephrotic syndrome have been shown to have very high Lp(a) concentrations independently of apo(a) isoform size (38, 42, 50–56). Furthermore, in patients with proteinuria or the nephrotic syndrome, decreases in Lp(a) levels have been reported with anti-proteinuric therapies or when the underlying nephropathy goes into remission (and proteinuria consequently reduces) (50, 54, 57–59). The underlying reasons for this relationship are unclear but, in contrast to non-nephrotic kidney disease, the increase in Lp(a) associated with nephrotic syndrome is likely to be a result of a general increase in protein synthesis by the liver due to high urinary protein loss rather than decreased catabolism (42, 50, 51, 60). In a stable isotope study of five patients with the nephrotic syndrome versus five control subjects [with varying apo(a) isoform sizes], despite higher Lp(a) levels in the nephrotic patients, the fractional catabolic rate was comparable between the two groups, suggesting increased synthesis may be the cause of high Lp(a) in these patients (60). In addition to this, Doucet et al. (18) showed more apo(a) fragments in plasma and in urine in nephrotic patients versus normolipidemic controls, despite comparable fractional catabolic rates. It was also suggested that large apo(a) fragments may be passively filtered by the kidney through the glomerulus and removed extrarenally, whereas smaller fragments are actively secreted into the urine (18, 45). Lower plasma albumin level and reduced oncotic pressure may also contribute to the increase in Lp(a) levels in nephrotic patients (61). ESRD patients undergoing peritoneal dialysis also have elevated Lp(a) levels regardless of apo(a) isoform size, similarly resulting from an overproduction of Lp(a) due to the substantial protein loss associated with peritoneal dialysis (akin to nephrotic syndrome) (30, 42, 50, 51).

## Lp(a) LEVELS AFTER KIDNEY TRANSPLANTATION

In patients that undergo kidney transplantation, Lp(a) levels appear to decrease in those previously treated with peritoneal dialysis independent of apo(a) isoform size and in patients previously treated with hemodialysis with large apo(a) isoform size, as might be anticipated given the relationship between apo(a) isoform size and Lp(a) levels with different dialysis modalities described above (62–65). Rapid decrease in Lp(a) after transplantation is consistent with a metabolic role of the kidney in Lp(a) catabolism and with the notion that the Lp(a) changes observed in CKD are due to loss of functioning renal tissue (62, 66, 67). Rosas et al. (66) examined 66 transplant patients with varying types of underlying renal disease and showed that Lp(a) levels were 35% lower 2 weeks after transplantation compared with Lp(a) levels prior to transplantation, and that a 50% lowering of creatinine was associated with 10% lowering of Lp(a). The association between Lp(a) levels and GFR (and with the level of albuminuria) in CKD is observed even after transplantation, which explains why, although Lp(a) levels are lower than among patients on dialysis, they may still remain slightly higher than normal after transplantation (68–70). These observations clearly demonstrate the nephrogenic origin of Lp(a) elevation in ESRD. Furthermore, Kostner et al. (71) observed higher Lp(a) levels and lower urinary apo(a) fragment excretion in a comparison of 116 transplant patients with normal or impaired kidney function versus 109 healthy controls. However, only patients with impaired creatinine clearance secreted less apo(a) than controls, suggesting excretion may be reduced after transplant in those with impaired excretory graft function, which may contribute to higher Lp(a) levels.

The role of immunosuppression therapy in influencing Lp(a) levels among transplanted patients has also been explored in previous studies with varying results (62, 65, 67, 72–74). Overall, the somewhat limited evidence suggests that the impact of kidney transplantation on Lp(a) levels is not strongly dependent on antirejection therapy.

## Lp(a) AND CARDIOVASCULAR OUTCOMES IN CKD

Cardiovascular disease is a major cause of death in patients with CKD (11). Patients with mild CKD are at risk of cardiovascular disease with a similar phenotype to the general population (i.e., typical atherosclerotic disease causing myocardial infarction and ischemic stroke), but the risk of heart failure increases more rapidly than the risk of atherosclerotic disease as eGFR falls. Nevertheless, lowering LDL-cholesterol with statin-based therapies reduces the risk of atherosclerotic events across the spectrum of CKD, albeit with smaller relative effects as eGFR declines and with little evidence of benefit in the small group of patients on dialysis (75, 76). Studies in non-CKD-specific populations have shown that increased Lp(a) levels are associated with increased risk of coronary heart disease and aortic-valve calcification, and Mendelian randomization studies suggest that these relationships are causal (5, 6). The possible mechanisms by which Lp(a) levels may cause coronary heart disease and aortic-valve calcification in non-CKD-specific populations; for example, the role of oxidized phospholipids in mediating risk through pro-inflammatory and pro-calcifying effects are also likely to be relevant to a CKD population and have been reviewed elsewhere (5, 77, 78).

Lp(a) levels in the general population are highly positively skewed, with an average of about 12 mg/dl, but with only 20% of individuals having Lp(a) levels above 50 mg/dl in Caucasian populations (79). In a case-control study of Lp(a), including 995 coronary heart disease cases and 998 coronary disease-free controls, the geometric mean Lp(a) levels were 3.4, 6.9, 10.2, 18.4, and 50.0 mg/dl among fifths of controls versus 3.1, 6.9, 10.6, 18.4, and 56.6 mg/dl among fifths of cases respectively (80). This and other studies have shown that increases in coronary disease risk are observed mostly in the 20–40% of individuals with the highest Lp(a) levels. For example, individuals in the top fifth of Lp(a) levels [and similarly in individuals in the fifth with smallest apo(a) isoform size] have shown a 2-fold higher risk of coronary heart disease compared with those in the bottom fifth of Lp(a) levels [or largest apo(a) isoform size] and, overall, about a 40% higher risk of incident cardiovascular disease per 1 SD higher Lp(a) level (SD = 32 mg/dl) (7, 80–83).

Various studies, many with limited statistical power, have examined whether Lp(a) contributes to increased cardiovascular risk in patients with CKD, but their conclusions have been inconsistent (84–92). Lp(a) elevations in non-nephrotic CKD are generally observed in individuals with larger apo(a) isoform sizes, and thus in those with lower genetically determined plasma Lp(a) levels. Based on results observed in non-CKD-specific populations, even a large relative increase in plasma Lp(a) in these individuals would be expected to yield only a small absolute Lp(a) difference and hence would have a correspondingly small effect on cardiovascular risk [e.g., doubling Lp(a) from 5 to 10 mg/dl would be expected to correspond to ∼5% increase in risk); this may explain a lack of consistency between the results of different studies. Furthermore, there are numerous potential biases in observational studies of patients with CKD, such as confounding by disease and treatment status (93–95), as well as potential Lp(a) assay-related biases, which may make the results of some studies less reliable and therefore need to be considered in the interpretation of the available data.

In the Cardiovascular Health Study, there was no significant association of Lp(a) with cardiovascular mortality among a subgroup of 1,249 CKD patients. However, the risk estimate was comparable to that observed in those without CKD in whom a significant association of Lp(a) with cardiovascular mortality was reported (91). Gault et al. (35) suggested that Lp(a) was not a risk factor for coronary disease in hemodialysis or peritoneal dialysis patients, albeit this was based on a study of only 52 hemodialysis and 58 peritoneal dialysis patients versus 56 controls. By contrast, other studies have suggested that higher Lp(a) levels and smaller apo(a) isoform sizes are independent risk factors for cardiovascular disease in hemodialysis patients (84, 86, 90, 96, 97). It has been postulated that prolonged residence time of Lp(a) in hemodialysis patients may contribute to the high risk of atherosclerosis in these patients (15). Kronenberg et al. (86) demonstrated that apo(a) phenotype was an independent predictor of coronary events in 440 hemodialysis patients, suggesting that small apo(a) isoform size was associated with about a 2-fold increased risk of atherosclerosis in these individuals. The apo(a) phenotype has also been shown to predict carotid atherosclerosis in ESRD patients (98). The 4D study of 1,255 hemodialysis patients with type 2 diabetes reported no association of higher ln Lp(a) levels or small apo(a) isoforms with combined cardiovascular events (hazard ratio, 1.04; 95% CI, 0.97–1.11; *P* = 0.30); however, significant associations of Lp(a) levels with all-cause mortality and fatal infections were reported overall, and particularly in patients ≤66 years of age (99). However, these associations were based on relatively small numbers of events, 599 deaths including 123 deaths from infection, and more data are needed to explore the relationship of Lp(a) with infection. More recently, the CRIC investigators have reported a study among nearly 4,000 CKD patients who were non-dialysis dependent at baseline, and among whom 815 had a myocardial infarction during the 7.5 years of follow-up. This study suggested a weakly statistically significant positive association of Lp(a) levels with risk of myocardial infarction (92). However, somewhat surprisingly, the event rate was lowest in the second quartile of Lp(a), not the first (postulated to be linked to the higher baseline triglyceride levels in the first quartile), resulting in additional uncertainties.

In summary, the available evidence in CKD populations suggests that, similarly to the general population, those with high Lp(a) levels are at increased risk of cardiovascular disease, although it remains unclear whether the increased risk is apo(a) isoform size dependent.

## POTENTIAL FOR THERAPEUTIC INTERVENTION IN PATIENTS WITH CKD

Observational and genetic evidence supporting the potential role of raised Lp(a) levels in the causation of coronary disease has generated considerable interest in Lp(a) as a potential drug target (5, 7, 81, 82, 100). However, there are no mainstream specific Lp(a)-lowering therapies currently available. Lp(a) can be lowered by some known cardiovascular therapies, but the impact of this on cardiovascular outcomes is unclear and potentially small. For example, as well as increasing HDL-cholesterol and lowering LDL-cholesterol, niacin lowers Lp(a) levels by 20–30%, likely as a result of reducing Lp(a) production (79, 101–103). Large-scale randomized trials in populations at high vascular risk have shown there to be no significant benefits of extended-release niacin formulations (in addition to statin therapy) on vascular outcomes and have also demonstrated significant hazards (104, 105). Additional Lp(a) data will soon emerge from the THRIVE trial (104) and will help to further elucidate the relationship between niacin-laropiprant, Lp(a) levels, and apo(a) isoform size. CETP inhibitors (106, 107) and PCSK9 inhibitors (108–111), as well as mipomersen (112), have also been shown to lower Lp(a) levels. In the REVEAL randomized trial of the CETP inhibitor, anacetrapib, which has large effects on HDL-cholesterol and non-HDL-cholesterol, Lp(a) levels were reduced by an average of 25%. However, the risk reduction in coronary death or myocardial infarction was consistent with the effects anticipated from the difference in non-HDL-cholesterol alone (113). Similarly, in the FOURIER trial, the effects of the PCSK9 inhibitor, evolocumab, on coronary death or myocardial infarction was consistent with the LDL-cholesterol lowering effect, despite also lowering Lp(a) by 27% (114). Lp(a) response dependencies on baseline Lp(a) levels and/or apo(a) isoform size may also limit their potential for additional related risk reduction. For example, the proportional Lp(a) reduction in response to evolocumab in the LAPLACE study varied considerably by baseline Lp(a) level, with larger absolute reductions, but smaller percent reductions, in those with higher Lp(a) versus those with lower Lp(a) at baseline (111). However, none of these trials were able to determine directly the effect of Lp(a) lowering on risk of cardiovascular events due to the considerable impact of each of these treatments on other lipid fractions, thus follow-up studies exploring the relevance of Lp(a) levels will be of considerable interest. Given these limitations, the recent development of an antisense inhibitor to apo(a) that can selectively reduce plasma levels of Lp(a) by up to ∼90% [relatively independently of baseline Lp(a) levels and apo(a) isoform size] has generated considerable excitement (78, 115–117). However, large-scale outcomes trials have yet to be performed in high-risk populations, including CKD patients, to assess the impact on disease outcomes. These trials will be the first to directly and fully assess the potential benefits of specific Lp(a) lowering on vascular risk.

Reducing the risk of cardiovascular disease among the general population and particularly those at highest cardiovascular risk, such as patients with CKD, is an area of significant unmet need. Designing clinical trials in populations with high Lp(a) levels, irrespective of CKD, would be beneficial when assessing the impact of Lp(a) lowering on cardiovascular events, because previous studies have shown increases in cardiovascular risk are mostly limited to individuals in the top one- to two-fifths of Lp(a) levels and are particularly associated with Lp(a) levels above 50 mg/dl (5). Although loss of kidney function increases Lp(a) levels [chiefly in individuals with larger apo(a) isoform sizes], previous data suggest that median Lp(a) levels remain below 30 mg/dl (2) and thus, in general, this increase is unlikely to result in a major additional contribution to cardiovascular risk. Therefore, despite the increase in both vascular risk and Lp(a) levels in CKD populations, trials of Lp(a)-lowering drugs in CKD patients (particularly those in nonprotein losing states), although potentially beneficial, would need to be very large to be sufficiently powered to detect clinically relevant effect sizes. Furthermore, patients with nephrotic syndrome or those treated by peritoneal dialysis, in whom elevated Lp(a) levels are independent of apo(a) isoform size, are similarly an unpromising population for study, because nephrotic syndrome is often transient and patients do not remain on peritoneal dialysis for many years (before they are transplanted or treatment failure requires a change in modality to hemodialysis).

## CONCLUSIONS

Various studies have shown that kidney function is one of the few nongenetic factors known to influence plasma Lp(a) levels, and support a role of the kidney in Lp(a) catabolism. Kidney disease results in pronounced changes in Lp(a) levels, with elevated Lp(a) levels in protein-losing states that are likely to be the result of increased synthesis, and elevated Lp(a) levels in non-nephrotic patients and patients treated with hemodialysis who have (genetically determined) large apo(a) isoform size, that are likely to be the result of reduced clearance. Kidney transplantation lowers Lp(a) levels, consistent with the acquired nature of the Lp(a) abnormality.
